# Utilization of Epilepsy Monitoring Unit by General Neurologists

**DOI:** 10.7759/cureus.27144

**Published:** 2022-07-22

**Authors:** Shahram Izadyar, Amr Ewida, Elena M Kleinhenz, Victoria Titoff

**Affiliations:** 1 Department of Neurology, State University of New York (SUNY) Upstate Medical University, Syracuse, USA

**Keywords:** emu referral, epilepsy monitoring unit, video-eeg, long-term monitoring, epilepsy

## Abstract

Background

Epilepsy monitoring unit (EMU) is a growing service that allows physicians to evaluate, diagnose, and manage epilepsy in a safe and cost-effective way. However, observations have indicated that the EMU is being underutilized by general neurology practice, possibly due to the lack of access and specific criteria known to all neurologists. There is limited data as of yet to support these observations. This study reviewed the rate of referral to the EMU from outpatient general neurology clinics at our institution.

Methods

In this retrospective study, records of 350 patients, 18 years or older with a diagnosis or diagnostic workup of epilepsy, managed by neurologists who did not specialize in epilepsy, were reviewed. We classified patients into three groups: ineligible for EMU referral, eligible and referred to EMU, and eligible but not referred to EMU based on six criteria namely characterization, classification, localization, determination of seizure frequency, medication adjustment, and differentiation between seizures and medication side effects.

Results

Our results demonstrated that 36.7% of patients who did meet the criteria were not referred to EMU. The most common criteria for patient referral in both groups, referred and not referred, was the characterization of seizures as epileptic or functional.

Conclusion

Our results show that EMU is underutilized by our general neurology clinics. Providing more information and increased awareness about criteria for long-term monitoring in EMU can improve the utility of this valuable tool and would be beneficial to patient care.

## Introduction

Epilepsy is one of the most common neurological disorders with an estimated lifetime prevalence of 7.60 per 1,000 persons around the globe [1]. In addition to obtaining a careful history, electroencephalogram (EEG) is a fundamental tool that provides valuable information that aids not only in diagnosis but also in monitoring of patients with epilepsy [2]. Given a low cumulative diagnostic yield of a routine EEG after a single seizure (39%) [3] and a low yield in capturing the paroxysmal events in question, video-EEG (VEEG) monitoring has become an essential part of epilepsy workup and management. The diagnostic yield of VEEG in detecting interictal epileptiform discharges and capturing paroxysmal events in question has been reported to be around 73%-88% [4-6] depending on the defined criteria of diagnostic yield and patient selection.

The utility of modern VEEG has been expanding since its advent in the 1970s, and by the 1980s, many centers have been using that for multiple purposes [7]. Currently, the recommended indications for VEEG monitoring range from differentiating epileptic seizures from functional seizures and other paroxysmal episodes with nonepileptic etiologies, such as sleep disorders, movement disorders, syncope, migraine attacks, and transient ischemia attacks, to seizure classification and medication adjustment, among others [6,8]. Proper utilization of VEEG can have important implications for patient care. Significant changes in diagnosis, classification, and management of seizures following VEEG, ranging from 44% to 83%, have been reported in several studies [5,9-11]. The yield of VEEG can be even higher when the antiseizure medications are withdrawn during the study [12]. The purpose of this study was to investigate the proper utilization of VEEG among neurologists who did not specialize in epilepsy.

This article was previously presented as a meeting abstract at the 2021 American Epilepsy Society Annual Meeting on December 5, 2021.

## Materials and methods

We retrospectively reviewed medical records and all notes related to neurology clinic visits of 350 patients above 18 years of age within a span of five years from January 2015 to January 2020 who were exclusively followed by general neurologists at outpatient clinics at our institution for diagnostic workup or management of epilepsy. None of these patients had a history of management by or referral to a neurologist with subspecialty training in epilepsy or epilepsy-focused clinical neurophysiology (epilepsy track) or with epilepsy-focused outpatient practice. We identified if each patient met at least one of the criteria listed in Table 1 during the study period for referral to our center’s epilepsy monitoring unit (EMU). We excluded patients who had been previously referred to EMU for the same criteria prior to the start of the study period. The presence of criteria was based on the perception of the treating neurologist as could be determined from their documentation and we, as the authors, avoided our own assessment. There was no significant change in the number of general neurologists and EMU availability at our institution during that timeframe.

**Table 1:** Criteria for referral to the epilepsy monitoring unit for video-EEG monitoring

Criteria	Explanation of criteria
Characterization	If there was uncertainty about the nature of paroxysmal events as epileptic or functional
Classification	Classification of epilepsy or epilepsy syndrome if it potentially could impact management
Localization	For the purpose of presurgical workup in cases of medically refractory epilepsy as a starting point of referral to an epileptologist and in cases that a referral to an epilepsy clinic for the same purpose had not been made
Determination of seizure frequency	In cases where the history was inadequate or inconsistent and if it could potentially impact management
Medication adjustment	When rapid switch or adjustment of medications was needed in a way that would be unsafe to do in an outpatient setting
Differentiation between seizures and medication side effects	In cases where history was inadequate or inconsistent

We continued to review the medical records in ascending order from the lowest medical record number until a total of 350 patients were included. We then stratified the included patients into three groups based on their eligibility for EMU referral according to the mentioned criteria: Group A, patients who did not meet the criteria and were not referred to EMU; Group B, patients who met the criteria and were referred to EMU including cases that the referral was declined by the patient or insurance; and Group C, patients who met the criteria and were not referred to EMU.

The State University of New York (SUNY) Upstate Medical University Institutional Review Board (IRB) has determined this project is exempt from IRB review according to federal regulations (IRB project number: 1688769-2).

Statistical analysis

Categorical variables were presented as numbers and percentages, and continuous variables were presented as means and standard deviations. Either one-way analysis of variance (ANOVA) or Pearson chi-square tests were used to evaluate the difference between groups. Statistical analyses were performed with WINKS Statistical Data Analytics (SDA) software (TexaSoft, Cedar Hill, TX) version 6.09 or R software (R-foundation) version 4.0.2, and p-values < 0.05 were considered statistically significant. The data are stored as de-identified participant data, which are available on reasonable request to the corresponding author.

## Results

**Figure 1:**
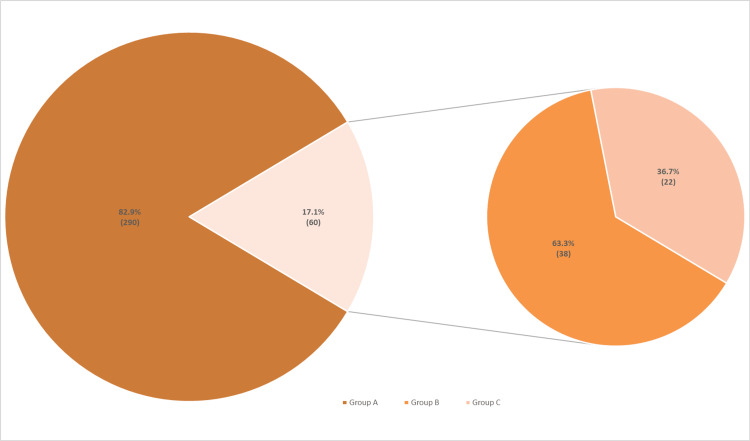
Proportion of patients who were referred versus those who were not referred to the EMU Large pie chart: Proportion of patients who did not meet the criteria for EMU referral versus those who did. Small pie chart: Proportion of patients who met the criteria for EMU referral and were referred versus those who were not referred. EMU: Epilepsy monitoring unit.

Of the reviewed records, 290 patients did not meet the criteria for referral to EMU and were not referred (Group A). Among the remaining 60 patients who met at least one of the criteria for referral, 38 (63.3%, Group B, 10.9% of total) were referred, and 22 (36.7%, Group C, 6.3% of total) were not referred to EMU (Figure 1).

Demographics and epilepsy classification of patients based on the 2017 International League Against Epilepsy (ILAE) classification in Groups B and C are shown in Table 2 where no statistically significant difference was seen in age, gender, underrepresented minority, language barrier, and epilepsy classification between the two groups.

**Table 2:** Demographics and epilepsy classification of patients who met the criteria for referral to the epilepsy monitoring unit

	Group B (n = 38)	Group C (n = 22)	p-value	95% CI
Mean age at the time of last clinic visit (SD)	44.87 (13.52)	52.36 (15.14)	0.052	-15.31 to 0.31 (of mean difference)
Male (%)/Female (%)	21 (55.3)/17 (44.7)	12 (54.5)/10 (45.5)	0.96	-
Underrepresented minority (%)	3 (7.9)	4 (18.2)	0.23	-
Language barrier (needing interpretation)	0	0	-	-
Classification of epilepsy			0.392	
Focal onset (%)	20 (52.63)	8 (36.37)		-
Generalized onset (%)	7 (18.42)	4 (18.18)		-
Unknown onset (%)	11 (28.95)	10 (45.45)		-

**Figure 2:**
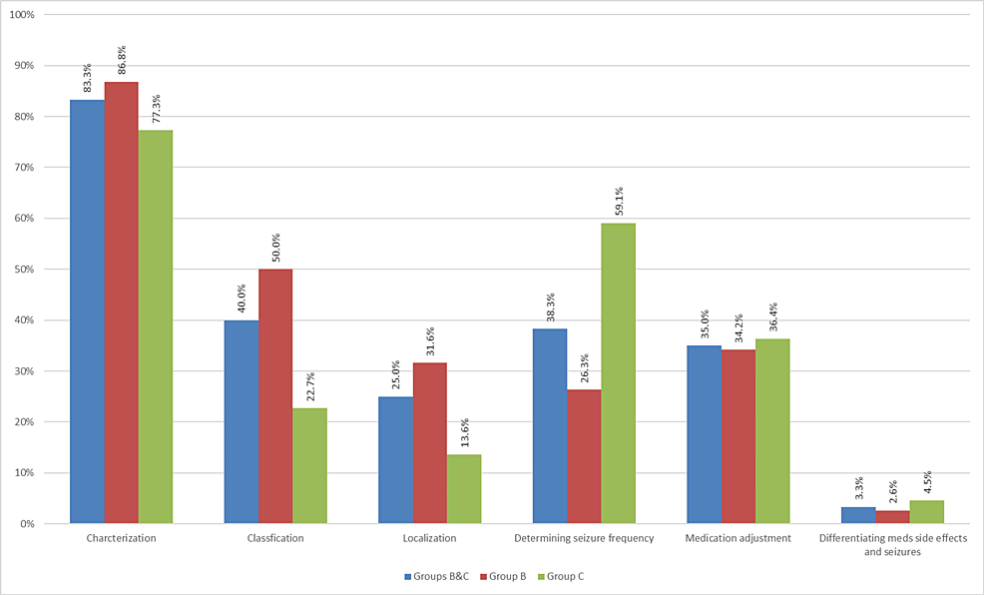
Prevalence of each referral criteria in Groups B and C individually and combined

**Figure 3:**
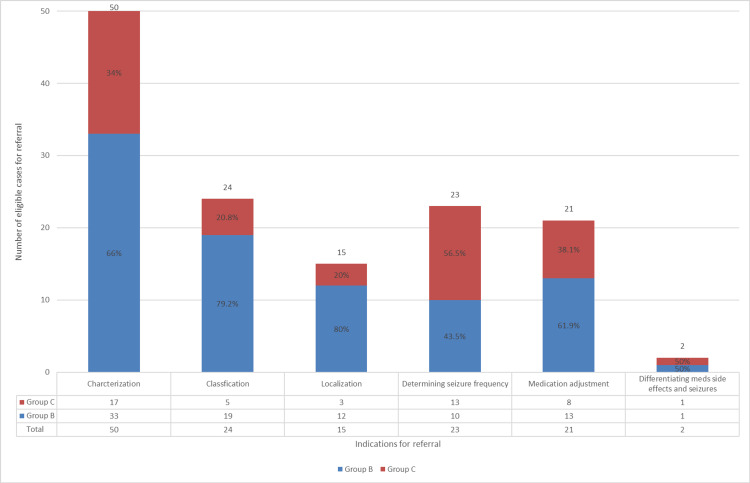
Proportion of patients in Groups B and C for each referral criteria

Thirty-one patients (81.6%) in group B and 15 patients (68.2%) in group C had more than one indication for EMU referral (p = 0.2). Characterization of the paroxysmal events as epileptic or functional was the leading criteria for EMU referral in both Groups B and C individually or combined. The prevalence of each referral criteria in Groups B and C is shown in Figure 2, and the proportion of patients in Groups B and C for each referral criteria is shown in Figure 3.

## Discussion

There are several studies that have evaluated the clinical utility of inpatient VEEG monitoring and its effect on diagnosis and management of patients with epilepsy, but data related to the utility of VEEG among general neurologists is scarce. Indications for VEEG monitoring have expanded in the past few years, and its utilization is considered to be cost-effective [13]. In our study, we adapted a list of criteria published by Shih et al. in 2018 [8] to determine the eligibility criteria for VEEG monitoring. We omitted the criteria for the ictal single-photon emission computerized tomography (SPECT) study from the original table since referral for that purpose is part of epilepsy surgical workup, which is not usually performed by general neurologists.

We found that 17.1% of our studied patients met at least one criteria for VEEG monitoring, and approximately, one-third of them were not considered for EMU referral by the treating general neurologists. The highest rate of non-referred criteria was "determining seizure frequency" followed by "differentiating between seizures and medication side effects." However, the number of patients in the latter group was very small and difficult to draw conclusions from. The lowest rate of non-referred criteria was seizure localization (for the purpose of presurgical evaluation, 20%) or seizure classification (20.8%). The delay between the first clinic visit and referral for epilepsy surgery programs has been identified in several reports from different countries: Mumford et al. found a delay of an average of 38.8 weeks in the New South Wales region in Australia [14]; in one study from Sweden, missed referral for epilepsy surgery was found to be 60 per 100,000 inhabitants, but most of those patients had not been seen by a neurologist [15], and similar delays have been reported from various other countries including United States [16].

Given that previous data suggest the course of diagnosis or management of 44%-83% of patients is affected by VEEG monitoring, our findings suggest that approximately 16.1%-30.5% of our study group who met the criteria for VEEG monitoring may have missed the opportunity to have optimization in diagnosis or management.

On the other hand, our findings about referred indications show that characterization of paroxysmal events was the leading criterion in patients who were referred to EMU. This finding is in line with other studies where characterization for diagnostic purposes was the leading proportion of referrals to the EMU [5,11]. At the same time in this study, one-third (34%) of patients with uncertainty about the nature of their paroxysmal events were not considered for the diagnostic gold standard, VEEG monitoring. Notably, about two-thirds (64.7%) of this group met more than one criteria for EMU referral. The reason for not utilizing VEEG in some patients in this group was documented as a partial response to a trial of antiseizure medications. Kerr et al. found that 17% of patients with functional seizures who had tried at least one antiseizure medication showed some response to the medication [17]. However, we cannot be certain about the proportion of responders to medications in our study who in fact had functional seizures. The above probably suggests that diagnosis of functional seizures may be delayed in some patients in this group. The average delay in diagnosis of functional seizures was reported to be 7.2 years (± 9.3 years) in a study published in 2002 [18] and slightly shorter in a more recent study published in 2016 with a mean duration of 5.6 years (± 8.2 years) [19]. We believe that increased awareness and available information about functional seizures in the past two decades as well as increased availability of VEEG have contributed to the decrease in delayed diagnosis.

The available data specifically about other criteria are sparse to compare with our findings. This study has some limitations: (a) Our data is limited to only a small group of patients at a single institution, which can introduce selection bias, and the generalizability of the findings can be limited; (b) as a retrospective study, the accuracy of the data depends on the extent and accuracy of the documentation in the records. For example, a neurologist may have discussed the referral with a patient who declined, but the reason for non-referral was not documented properly in the records; and (c) our data does not include the cost-effectiveness of the utilization of EMU in our study group.

## Conclusions

In the past few years, indications for VEEG monitoring have expanded, and this tool has also been considered to be cost-effective. Despite increased awareness and availability of VEEG monitoring, this valuable tool still seems to be underutilized among general neurologists. This can result in missing opportunities to optimize the diagnosis or management of patients with epilepsy such as delayed diagnosis of functional seizures or delayed referral for epilepsy surgery programs in eligible patients. Increased awareness among general neurologists about the criteria for long-term monitoring in EMU can improve the utility of this valuable tool and have a positive impact on patient care. In this effort, developing a standard set of widely known criteria for long-term monitoring in EMU and providing more information to general neurologists would be beneficial.

## References

[1] Fiest KM, Sauro KM, Wiebe S (2017). Prevalence and incidence of epilepsy: a systematic review and meta-analysis of international studies. Neurology.

[2] Tatum WO, Rubboli G, Kaplan PW (2018). Clinical utility of EEG in diagnosing and monitoring epilepsy in adults. Clin Neurophysiol.

[3] Baldin E, Hauser WA, Buchhalter JR, Hesdorffer DC, Ottman R (2014). Yield of epileptiform electroencephalogram abnormalities in incident unprovoked seizures: a population-based study. Epilepsia.

[4] Benbadis SR, O'Neill E, Tatum WO, Heriaud L (2004). Outcome of prolonged video-EEG monitoring at a typical referral epilepsy center. Epilepsia.

[5] Ghougassian DF, d'Souza W, Cook MJ, O'Brien TJ (2004). Evaluating the utility of inpatient video-EEG monitoring. Epilepsia.

[6] Cho YW, Motamedi GK, Kim KT (2019). The clinical utility of non-invasive video-electroencephalographic monitoring has been diversifying. Neurol Sci.

[7] Binnie CD, Rowan AJ, Overweg J, Meinardi H, Wisman T, Kamp A, da Silva FL (1981). Telemetric EEG and video monitoring in epilepsy. Neurology.

[8] Shih JJ, Fountain NB, Herman ST (2018). Indications and methodology for video-electroencephalographic studies in the epilepsy monitoring unit. Epilepsia.

[9] Chen LS, Mitchell WG, Horton EJ, Snead OC 3rd (1995). Clinical utility of video-EEG monitoring. Pediatr Neurol.

[10] Kumar-Pelayo M, Oller-Cramsie M, Mihu N, Harden C (2013). Utility of video-EEG monitoring in a tertiary care epilepsy center. Epilepsy Behav.

[11] Alving J, Beniczky S (2009). Diagnostic usefulness and duration of the inpatient long-term video-EEG monitoring: findings in patients extensively investigated before the monitoring. Seizure.

[12] Kirby J, Leach VM, Brockington A, Patsalos P, Reuber M, Leach JP (2020). Drug withdrawal in the epilepsy monitoring unit - the patsalos table. Seizure.

[13] Baheti NN, Radhakrishnan A, Radhakrishnan K (2011). A critical appraisal on the utility of long-term video-EEG monitoring in older adults. Epilepsy Res.

[14] Mumford V, Rapport F, Shih P (2019). Promoting faster pathways to surgery: a clinical audit of patients with refractory epilepsy. BMC Neurol.

[15] de Flon P, Kumlien E, Reuterwall C, Mattsson P (2010). Empirical evidence of underutilization of referrals for epilepsy surgery evaluation. Eur J Neurol.

[16] Jetté N, Sander JW, Keezer MR (2016). Surgical treatment for epilepsy: the potential gap between evidence and practice. Lancet Neurol.

[17] Kerr WT, Janio EA, Le JM (2016). Diagnostic delay in psychogenic seizures and the association with anti-seizure medication trials. Seizure.

[18] Reuber M, Fernández G, Bauer J, Helmstaedter C, Elger CE (2002). Diagnostic delay in psychogenic nonepileptic seizures. Neurology.

[19] Asadi-Pooya AA, Tinker J (2017). Delay in diagnosis of psychogenic nonepileptic seizures in adults: a post hoc study. Epilepsy Behav.

